# Liquid metal cooled fast reactor thermal hydraulic research development: A review

**DOI:** 10.1016/j.heliyon.2023.e16580

**Published:** 2023-05-24

**Authors:** Kafui Tsoeke Agbevanu, Seth Kofi Debrah, Emmanuel Maurice Arthur, Edward Shitsi

**Affiliations:** a Department of Nuclear Engineering, School of Nuclear and Allied Sciences, University of Ghana, P.O. Box AE1, Kwabenya, Accra, Ghana; b Department of Computer Science, Ho Technical University, P. O. Box HP 217, Ho, Ghana; c Nuclear Power Institute, Ghana Atomic Energy Commission, P.O. Box LG 80, Legon, Accra, Ghana; d Nuclear Research Centre, National Nuclear Research Institute, Ghana Atomic Energy Commission, P.O. Box LG 80, Legon, Accra, Ghana

**Keywords:** Thermal hydraulics, Liquid metals, Heavy liquid metal coolants, Fast reactor facilities, Experimental facilities, Lead-cooled fast reactors

## Abstract

The growing interest in fast reactors demands further innovative technologies to enhance their safety and reliability. Understanding thermal hydraulic activities required for advanced reactor technology in design and development is key. However, knowledge of Heavy Liquid Metal (HLM) coolants technology is not mature. The liquid metal-cooled facilities are required experimental platforms for studying HLM technology. As such, efficient thermal hydraulic experimental result is important in the accurate validation of numerical results. In this vein, there is a need to closely review existing thermo-hydraulic studies in HLM test facilities and the test sections. This review aims to assess existing Lead-cooled Fast Reactor (LFR) research facilities, numerical and validation works and Liquid Metal-cooled Fast Reactor (LMFR) databases around the world in the last two decades. Thus, recent thermal hydraulic research studies on experimental facilities and numerical research that support the design and development of LFRs are discussed. This review paper highlights thermal hydraulic issues and developmental objectives of HLM, briefly describes experimental facilities, experimental campaigns and numerical activities, and identifies research key findings, achievements and future research direction in HLM cooled reactors. This review will enhance knowledge and improve advanced nuclear reactor technology that ensures a sustainable, secure, clean and safe energy future.

## 1 Introduction

Globally, the nuclear industry is researching a wide range of cutting-edge technologies for nuclear reactor architecture. The next generation of nuclear power facilities, called innovative or advanced reactors, would have enhanced safety features than today’s reactors and will be operational by 2030 [1].

Lead and Lead–Bismuth Eutectic (LBE) are among the most optimistic coolants and spallation targets for ADS [2]. Despite corrosion and polonium contamination issues, these Heavy Liquid Metals (HLMs) are still among the most practical coolant alternatives for upcoming reactors. Key concerns associated with experimental activities of Lead-cooled Fast Reactors (LFRs) employing HLMs as the working fluids are that, they require intensive research [3].

Researchers and organizations working together to develop novel nuclear reactor systems cooled by HLMs need to adequately understand their design and development technologies. Understanding thermal-hydraulic phenomena required for advanced reactor technology in design and development is key.

Multiple experimental facilities have been developed to enhance research and development technology of the innovative reactors in the areas of flow and heat transfer, system, core, pool and subchannel thermal hydraulics. To support experimental results, numerical analyses are required. System codes, subchannel codes and computational fluid dynamic (CFD) codes are also being produced to predict HLM coolants flow and heat transfer. Therefore, thermal hydraulic analysis of such reactors is critical and experiments and numerical simulations must work together in this regard [4].

This review paper focuses on the thermal-hydraulic research activities performed with experimental test facilities to aid in the development and granting of licences of HLM cooled research reactors. The review aims to assess existing LFR research facilities, numerical and validation works and Liquid Metal-cooled Fast Reactor (LMFR) databases around the world in the last two decades. The specific objectives are: to identify existing LFR design issues and objectives for HLM coolant technology; highlight the current LFR experimental facilities, and research reactors and give a brief description of various experimental facilities; discuss various thermal hydraulic research activities undertaken, flow and heat transfer, numerical exercises and benchmark efforts and related research efforts in experimental facilities using HLM coolants; and enumerate the key findings, achievements and future research directions of advanced fast reactors using HLM coolant technology for research and development as well as joint research that could speed up and lower the cost of developing new technology.

## 2 Design issues and objectives of LFR development

In the GEN-IV reactors, sodium and Pb/LBE coolants are viewed as short- and long-term solutions [5]. An overview of LFR design and the development of related technologies by the Italian National Agency for New Technologies, Energy and Sustainable Economic Development (ENEA) is presented [6]. Pure lead (Pb) and LBE, have extremely high boiling temperatures [7], allowing the fast reactor to run at normal atmospheric pressure and preventing coolant boiling.

To accomplish the design and aid pre-licensing, Research and Development (R&D) efforts are required. Therefore, the construction of the appropriate system can be done after noting the gaps in technology that depend on the design and represent major subjects for LFR/Accelerator Driven Systems (ADS) advances. Several technological issues and objectives with LFR development are detailed in Ref. [8].

The concepts of LFRs have been initiated in several International Atomic Energy Agency (IAEA) member countries including Russia, Japan, South Korea, China, the EU, and the USA [9,10]. However, the success of these fast reactor concepts hangs on detailed research that borders on experimental data availability and appropriate numerical codes, methods, and technology that supports their design and development enhancement for safety and economic competitiveness. Lorusso et al. [11] discuss the current state and prospects of GEN-IV LFR development, highlighting ENEA's skills and expertise in GEN-IV LFR technologies.

Three categories of important issues and their respective objectives [8] have been highlighted in Table 1.

**Table 1:** Summary of LFR thermal-hydraulics issues and objectives.

No.	Issue	Objective
1	Component and system	Investigate and test the following: - Various forced convective flows (such as mixing, stratification, and oscillations at the surface) - The transition to buoyant flow - Natural circulation flow (such as pressure drop) - The interaction of fluid and structure - Fatigue from heat - Sloshing caused by seismic events
2	Fuel assembly thermal-hydraulic parameters	Investigate and test: - Forced and natural convection and transition - Subchannel flow spread (open wrap and wrap-less) - Cladding temperature with hot spots - Fuel assembly bow - Pressure-drop - Grid-rod-fretting - Interaction of fluid and structure - Vibrations resulting from flow
3	Integral tests on postulated and un-postulated accidents	Investigate the following: - Events and processes of the system with design, safety and operation - Flow blockage with related experimental/modelling studies - Accident management procedures - Component testing - Wide range of accident simulations and analyses - Scaling issues - Database generation to support the licensing process - Code assessment and validation

## 3 Research reactors and experimental facilities cooled with HLM

### 3.1 HLM research reactors

Table 2 provides a list of some member countries' thermal-hydraulic research and development efforts in support of LFRs [12]. The LFR research reactors' specifics are described in the [13,14]. The development of the demonstration reactors BREST-OD-300, MYRRHA, ALFRED, and CLEAR-I is currently underway to demonstrate Technology Readiness Level 7 of HLM nuclear system technology, achieve high safety standards, improve non-proliferation resistance, evaluate the economic viability of LFR technology, including high load factors, show better resource utilization by closing the fuel cycle, and validate the material selection.

**Table 2:** List of existing LFR research reactors.

Acronym	Type	Country	Coolant	Design Status	Purpose
ALFRED	LFR	EU	Pb	Being designed	Demonstration
BREST-OD-300	LFR	Russia	Pb	Being designed	Demonstration
CLEAR-I	LFR	China	LBE	Conceptual planning	Experimental
ELECTRA	LFR	Sweden	Pb	Being developed	Demonstration
ELFR	LFR	EU	Pb	Conceptual planning	Demonstration
G4M	LFR	USA	LBE	Being designed	Commercial
LFR-AS-200	LFR	Luxemburg	Pb	Conceptual planning	Commercial
MYRRHA	LFR	Belgium	LBE	Being designed	Experimental
PEACER	LFR	Rep. of Korea	LBE	Conceptual planning	Demonstration
SEALER	LFR	Sweden	Pb	Conceptual planning	Commercial
SVBR-100	LFR	Russia	LBE	Being designed	Commercial
W-LFR	LFR	USA	Pb	Conceptual planning	Demonstration

### 3.2 HLM experimental facilities

This section tabulates experimental facilities that are currently in use to promote thermal hydraulic research and the development of novel reactors using HLM coolants, as shown in Table 3 extracted from the IAEA database LMFNR [12].

**Table 3:** Lead-based thermal hydraulics experimental facilities.

Facility Name	Reactor Type	Country	Max. Power (kW)	Coolant	Max. Operating Temperature (^o^C)	Max. Operating Pressure (MPa)
COMPLOT	LFR	Belgium	75	LBE, Air	400	16
E-SCAPE	LFR	Belgium	100	LBE, Air, Other	320	0.7
CLEAR-S	LFR	China	3500	LBE	500	2
KYLIN II TH FC	LFR	China	300	LBE	400	1.2
KYLIN II TH MC	LFR	China	500	LBE	500	1.2
KYLIN II TH NC	LFR	China	24	LBE	500	1.0
ELEFANT	LFR	Germany		Lead	500	
THEADES	LFR	Germany	500	LBE	450	1.0
THESYS	LFR	Germany	20	LBE	400	0.5
CIRCE-HERO	LFR	Italy	1000	LBE	120	0.45
CIRCE-SGTR	LFR	Italy	30	LBE	350	1.6
HELENA	LFR	Italy	250	Lead	480	1.0
NACIE	LFR	Italy	235	LBE	150	1.0
LIFUS	LFR	Italy	90	LBE, Other	220	20
JLBL-3	LFR	Japan	41	LBE	450	0.5
JLBL-4	LFR	Japan		LBE	500	0.5
IMMORTAL	LFR	Japan		LBE	500	0.5
HELIOS	LFR	South Korea	60	LBE	350	1.0
IPUL	LFR	Latvia	20	LBE	450	1.0
6B	SFR, LFR,	Russia	1200	Sodium, Other	450	0.6
TALL3D	LFR	Sweden	80	LBE	460	1.0
DELTA	LFR	USA	50	LBE	100	0.7

### 3.3 Brief descriptions of some HLM experimental nuclear facilities

**Fig. 1:**
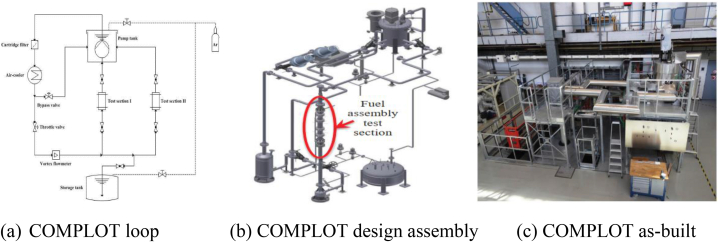
(a) COMPLOT loop [15,16], (b) design assembly [16] and (c) as-built diagrams [16].

A complete hydraulic flow model to study the MYRRHA core is created using the large-scale isothermal loop known as COMPLOT [15], which enables characterizing full-scale MYRRHA components in flowing LBE [16]. A high flow rate of 36 m^3^/h at which LBE can be pumped through the system was adopted. This isothermal loop can support varying temperatures of up to 400 °C in a test section to assess the impacts of temperature. The fuel assembly and other components form part of the COMPLOT system. System and CFD codes can both be evaluated with COMPLOT. A diagram of the COMPLOT loop and its components is shown in Fig. 1(a–c), as well as the COMPLOT LBE facility's design assembly and as-built state.

**Fig. 2:**
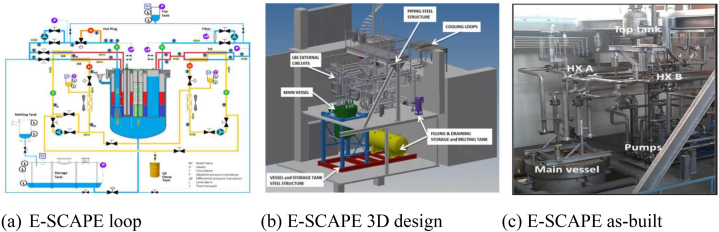
(a) The E-SCAPE loop [18], (b) 3D design [17] and (c) as-built diagrams [18].

E-SCAPE is 1/6th the size of MYRRHA research infrastructure, and it contains the European SCAled Pool Experiment; a thermohydraulic and electrical core simulator model [17]. It will provide experimental information for the designers to consider when generating flow paths for both forced and natural circulation. Additionally, the LBE computational techniques can be confirmed. Fig. 2(a–c) shows various diagrams of the E-SCAPE facility [[17], [18], [19]].

**Fig. 3:**
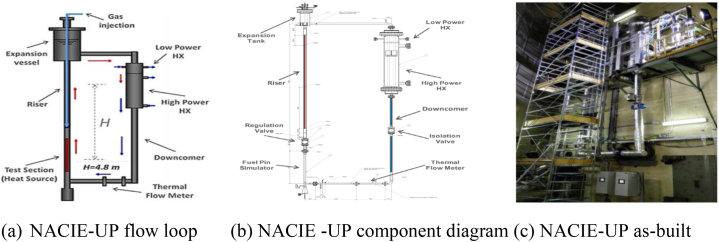
(a) NACIE-UP loop [22], (b) component diagram [20] and (c) as-built facility [21].

Several experimental facilities have been developed at ENEA Brasimone Research Centre, in Italy, to undertake integral circulation experiments, pool thermal-hydraulic investigations, and heat transfer studies in fuel rod bundles. New parts were being added to the facility, currently known as NACIE-UP. Fig. 3(a–c) depicts the NACIE-UP facility, which is rectangular and enables researchers to carry out experimental activities in thermal-hydraulics, fluid dynamics, and heat transfer among others, as well as obtain critical correlations to aid nuclear reactor design that uses HLM coolants [[20], [21], [22]]. On NACIE, a few test campaigns with simpler Fuel Pin bundle Simulator (FPS) and components were run. As part of recent development, a basic FPS made up of 19 wire-spaced pins has just been added. A test campaign was run in 2016 and 2017 using the same bundle and new parts in the loop, and a prototype thermal mass flow meter was created and made by ENEA and Thermocoax. The focus of the new campaign has been transient tests, notably the switch from high power and forced circulation to natural circulation at low FPS power [22]. A comprehensive heat transfer assessment is guaranteed with the provision of necessary instrumentation within the fuel bundle both at a steady state and during transients.

**Fig. 4:**
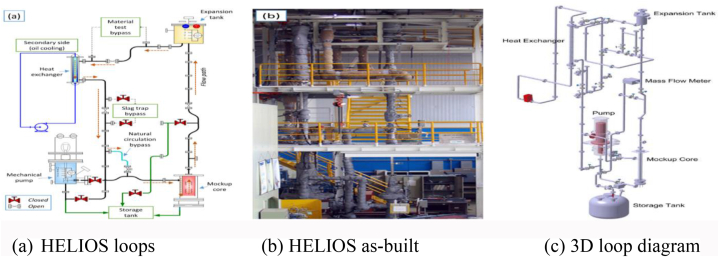
(a) HELIOS loop [23] and (b) as-built facility [23], (c) 3D loop diagram [24].

Fig. 4(a–c) shows a thermal-hydraulic scaled-down facility called HELIOS for PEACER-300 [23,24]. HELIOS is made up of two closed loops that model the PEACER-300 reactor's primary and secondary sides, respectively.

The PEACER-300 design has five thousand to-one in terms of thermal power and one-to-one in terms of height and is intended to be tested for operability, safety, and suitability for thermal-hydraulic experiments in either forced or natural circulation modes, as well as materials corrosion testing. The height and total pressure loss coefficient of HELIOS were identical to those of the prototype.

**Fig. 5:**
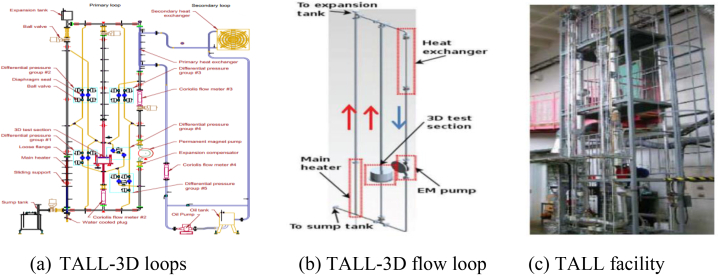
(a) TALL-3D loop [26] (b) flow loop [28], and (c) facility [25].

The TALL-3D facility in Fig. 5(a–c), is primarily intended to provide information for CFD and the system thermal-hydraulic (STH), as well as STH-CFD linked codes [[25], [26], [27], [28]]. Several safety-related transients with transitions between them can be tested at the facility, along with forced, natural, and mixed circulation regimes. The facility's layout promotes two-way feedback between the dynamics of the loop system and local 3D flow phenomena including temperature stratification and mixing inside a test section resembling a pool.

The TALL facility is an averaged-sized experimental structure at KTH in Sweden, constructed to assess the thermal and hydraulic efficiency of reactors cooled with LBE, as well as to support the European Transmutation Demonstration using ADS cooled with LBE [29]. The TALL-3D which was developed from the TALL facility recently conducted a test to investigate the following: validation of linked CFD-STH codes using feedback between STH and the 3D effect; pre-test analysis for validation of solidification models using LBE solidification events and the effect of flow structure [30]; validation of STH codes using loop STH in various transients; and validation of CFD codes using mixing and stratification occurrences in a pool were performed. Also, a lead-cooled facility known as Metal Liquid Loop (Meliloo) was created and improved as part of a SESAME project at CVR, Research Centre REZ, Czech Republic, to conduct solidification tests and check the accuracy of CFD techniques [[31], [32], [33], [34]].

Worldwide, there are many different experimental facilities, each with its specific requirements, operating conditions, and working fluids. Some of these experimental facilities could be adjusted to fulfil specific needs despite their high initial expenditures. When commissioning new facilities for a specific use, consideration should be given to design elements that make operations simpler and flexible to adjust performance early in the design phase to fit development demands. Details of other experimental facilities and tests conducted not described in this review can be accessed in Ref. [12]. Worthy of mention is the recent design of SEALER by LeadCold Reactors for off-grid utilization, and its commercial usage in the mining sector in Canada, which encourages the marketing of various SEALER units [35,36]. Moreover, LeadCold, KTH, and Uniper Sweden have collaborated to build demonstration LeadCold SEALER small modular reactors (SMRs) at the Oskarshamn plant site by 2030. Additionally, SEALER LeadCold Reactors as well as Westinghouse Electric Company are developing LFRs in the UK [35,37], with Westinghouse Electric Company and Ansaldo Nucleare collaborating to further develop next-generation LFR technology already ongoing with lead-based test facilities in Italy, Romania, US and UK. Furthermore, the W-LFR program embarks on the creation of experimental investigation, model and computer code design and development stages of the safety analysis process [38,39].

## 4 Analysis of thermal hydraulics activities performed on HLM-cooled research reactors

Heavy liquid metals are becoming increasingly popular in several scientific and industrial domains. A meeting to theoretically and experimentally investigate HLM thermal hydraulics was arranged to create a global venue for sharing the most recent development of such knowledge. The main aim of the technical meeting was to analyse the current CFD codes employed in HLM modelling; propose initiatives in both numerical as well as experimental future research works [40]. The areas of interest identified for future R&D are in Table 4.

**Table 4:**
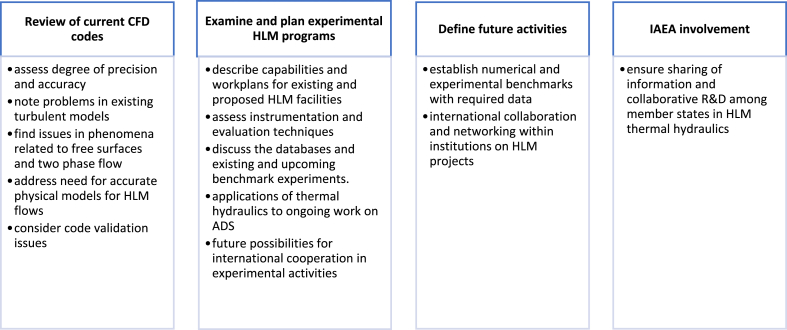
Identified areas for future HLM R&D.

Several essential crosscutting thermal-hydraulic concerns for advanced fast reactors were highlighted [41]. The European work on innovative thermal-hydraulic events for novel nuclear reactor systems has been provided [25] and the latest discoveries and efforts for developing LMFR thermal-hydraulic technology have been discussed [42,43]. The focus has been the thermal hydraulics in fuel assemblies, pools, and systems [7,44]. In this review paper, flow and heat transfer activities supported by experimental facilities cooled by HLM are categorized as the core; pool; STH and integral CFD simulations.

### 4.1 Core thermal hydraulics

A review of thermal hydraulics concerns in nuclear reactors was written by Ref. [45]. The goal of the hydrodynamic and thermal-hydraulic core analysis is to establish optimal operating parameters for the nuclear power plant that are both safe and cost-effective. Nuclear reactor core thermal-hydraulic analyses often use subchannel analytic approaches to predict various thermal-hydraulic safety limits [25,41]. Full-scale CFD study of the core is a challenging and time-demanding task because of the difficulty of rod bundle design, various turbulence scales, and computational resource limits. Subsequently, CFD examination of core thermal hydraulics in liquid metal reactors has been reported [42]. Three-dimensional CFD simulations using modern innovative computer power and tools, allow safety designers to acquire further comprehensive information related to heat transport in fuel assemblies that are cooled by liquid metals, backed up by the necessary experimental drive. In addition, the strategy of using a stable turbulent Prandtl number and the usual Reynolds analogy was adopted in the simulation of heat transfer validation, but [43] indicated the inefficiency of the method in the predictions of heat transport in liquid metals. As a result, [46] provided a recent evaluation of available approaches. Therefore, the simulations of deformed, vibrating, and blocked assemblies must be undertaken and validated by the nuclear community in the future.

For nuclear reactor cores, the prospects for sub-channel thermal hydraulic codes were reviewed [47]. The review examines several aspects of earlier rod bundle experimental, analytical, and computational research, as well as future directions resulting from previous research. A sub-channel code being built for an LBE fast reactor with numerous fuel assembly architectures was conducted [48]. An initial investigation of sub-channel thermal-hydraulic on 61 wire-wrapped bundles with LBE coolant was undertaken [49]. They noted the necessity of detailed thermal-hydraulic assessment of varying mass flow rates as well as heating powers. Furthermore, the MYRRHA’s LBE-cooled wire-wrapped fuel assembly was tested thermally and hydraulically [50].

More so, the fuel assembly interior blockage analyses of the NACIE-UP facility were conducted using CFD [51]. The blockage can be identified by placing Resistance Temperature Detectors in the mixing region. To properly study various flow regimes of the interior blockage, experiments relevant to the Blocked Fuel Pin bundle Simulator (BFPS) test section were conducted. The findings suggest that the various codes produce similar outcomes when applied to the mixing model, even though the exact values may be different. To obtain a more comprehensive comparison, an advanced Large Eddy Simulation (LES) calculation should be performed and additional temperature data from NACIE-UP at ENEA should be used as a benchmark. The result aims to provide experimental data to aid in the development of the ALFRED. Consequently, different modelling approaches in Reynolds Averaged Navier-Stokes (RANS), Unsteady-RANS (URANS), and LES were compared (e.g., meshing, turbulence modelling, programs, and users).

ENEA conducts experimental testing to support the ALFRED reactor to imitate accident scenarios [52]. Moreover, a thermal-hydraulic code for subchannels ANTEO+ was created for the simulation of pin bundle layouts using liquid metal coolants [53]. Because ANTEO+ is based on empirical correlations, the validation step is critical. In addition, the temperature distribution in the CIRCE facility's pin bundle was examined [54]. Furthermore, heat transmission in an FPS that is cooled with HLM was examined in the NACIE-UP facility [55]. As many as 67 thermocouples were installed in the fuel assembly for tracking temperature distribution in various sub-channels of the flow.

A CFD benchmark for an HLM fuel assembly is presented by Ref. [56]. Widely spread wire-spaced fuel assembly provides a difficult architecture with limited flow data available to perform verification and validation analyses using CFD simulations, according to the researchers. Conjugate heat transfer in the fuel rod as well as wire-spaced cladding is used to mimic a 19-pin fuel assembly with characteristics similar to MYRRHA. Also, Argonne National Laboratory working with Dutch Research and consultancy Group, Belgian Nuclear Research Centre (SCK-CEN), and Ghent University performed and compared a sequence of computations for the fuel-pin bundles that make up a fast reactor core with the International Nuclear Energy Research Initiative (I-NERI) of the US Department of Energy [57]. The CFD code findings using the SHARP package and STAR-CCM+ commercial software proved to be advantageous as a consequence of the blind benchmark exercise.

The thermal-hydraulic code for transient assessment of lead and LBE reactors was developed and validated [58]. The developed code LETHAC was utilized and the results were compared to information from the KYLIN-II experiment having a 61-fuel pin bundle. The preliminary validation work was undertaken to utilize experimental information from the KYLIN-II and TALL facilities. The comparison of the primary correlations and that of the KYLIN-II wire-spaced bundle was carried out. Consequently, the researchers are of the view that further assessment is required to improve understanding of more of such intricate and delicate occurrences in multidimensional lead pools and multiphase events. Therefore, greater effort should be invested in validating these new models using more specific experimental data. Additionally, [59] performed a comprehensive study of the friction coefficient of a wire-wrapped fuel bundle and recognized the comprehensive Cheng and Todreas (CTD) correlation reported in 1986 as the most often utilized relationship for the development and safety assessments of Generation IV Sodium-cooled Fast Reactors. The CTD correlation has been revised and upgraded based on crucial new information [60].

Lyu et al. [61] looked at the thermal-hydraulic characteristics of a wire-wrapped fuel bundle with a reduced wire diameter of peripheral rods using a numerical model. Using RANS-based CFD simulation, a comparison between the novel bundle and the standard one was conducted to examine the new bundle heat transfer and fluid flow performance. Additionally, the impact of Inter-Wrapper Flow (IWF) on fuel assemblies with liquid metal cooling was investigated experimentally [62,63]. Furthermore, the CFD approach on a 19-pin wire-spaced fuel assembly using LBE was scaled to test the MYRRHA reactor’s fuel assembly [64]. The numerical model created was heavily utilized to simulate the obstructed assembly flow with a cylindrical blockage and was validated against experimental data.

In an experimental study at ENEA, the NACIE-UP facility has installed a fuel pin package simulator with varied power distribution to mimic the primary coolant's mass flow rate progression from forced to natural convection [26], and how local temperatures and perhaps system behaviour is affected by the power distribution were found out [52]. The experimental results proved helpful in determining the bundle’s characteristics and calculating the heat transfer coefficient. Importantly, the NACIE-UP benchmark was developed as part of the EU SESAME Project to improve, develop, and validate current thermal–hydraulic codes for HLM systems [65].

A fully developed turbulent flow study conducted at the NACIE-UP facility is reported [66]. The Baseline Algebraic Reynolds Stress model employed in ANSYS CFX can perform accurate forecasting of bundle flow in comparison to Direct Numerical Simulation data and other turbulence models. Therefore, model experiments are required to comprehend physics, validate experimental instruments, and qualify new designs for licensing.

Again, the pumping failure effect on flow and temperature distribution was performed [67] using ANSYS FLUENT-15. In the CLEAR-S facility, transient 3D CFD simulations of thermal stratification were performed during a Protected Loss of Flow Accident (PLOFA). The study aids technology advancement of lead-based reactors considering the impact of thermal load on structural components of the reactor and endangering the stability and safety of the system.

### 4.2 Pool thermal hydraulics

Modern and competent numerical tools are required for engineering design, performance analysis, and safety evaluation in Generation IV HLM-cooled nuclear reactors. CFD and subchannel codes allow the extensive thermal-hydraulics study of pool-type nuclear reactors. Jeltsov et al. [68] used a TALL-3D experiment to validate a CFD code Star-CCM+ for liquid LBE thermal hydraulics. Star-CCM+ was utilized for a computational simulation of an experiment comparing forced to natural flow in TALL-3D. To assess the validity of the CFD model, computational and experimental data are compared. Furthermore, a review of the initial set of experimental tests performed in the CIRCE HLM large pool to study mixed convection and stratification processes was conducted [69]. They conducted thermal stratification in LFR pool reactor studies both analytically and experimentally utilizing Loss of Flow (LOF) and Protected Loss of Heat Sink (PLOHS) simulation. The design of GEN-IV LFRs is supported by research on thermal hydraulics processes that is both numerical and experimental.

An accident resulting from a Heat eXchanger Tube Rupture (HXTR) known as the Steam Generator Tube Rupture (SGTR) accident, is significant and should be taken into account while designing and evaluating HLM-cooled reactors. Wang [70] investigates the evolution of knowledge regarding the HLM-cooled reactor HXTR postulated accident. It was noted that the HXTR disaster should be addressed first by upgrading the pertinent experimental infrastructure and numerical tools. Furthermore, [71] investigated the consequences of an SGTR accident, capable of causing LBE to react with highly pressurized water; to assist in the development and evaluation of steam generators concerning design and safety in the CLEAR project. Lorusso et al. [72] used CIRCE-HERO to evaluate MYRRHA primary heat exchangers in an experimental simulation to enhance plant safety. Also, in the ELSY conceptual reactor, there has been a discussion of the potential for steam bubbles to migrate to the core and then accumulate as voids in the primary system [73].

Narcisi et al. [74] conducted an assessment of stratification in the CIRCE-ICE using the RELAP5-3D code and compared the results to experimental data. The code proved capable of accurately simulating operational conditions in the test section's primary flow channel. More so, forced and natural convection in a pool-type nuclear facility’s upper plenum was explored [75]. In a low flow rate condition with a high-temperature difference over the plenum height, barrel hole jets are much weaker, with no effective circulation in the plenum, resulting in thermal stratification.

In the CIRCE-HERO infrastructure, [76] report on the protected loss of primary pump transients during the pre-test. According to simulations, the HERO bayonet bundle has outstanding thermal-hydraulic performance in normal settings and also allows operation in natural circulation. Subsequently, in the same facility, [77] performed a PLOFA transient post-test simulation, an experimental campaign aimed at investigating HERO behaviour. For the test, the PLOFA event that replicates the primary pump closure is used with reactor scram, in addition to the Decay Heat Removal (DHR) system.

Thermal stratification particularly in HLM large pool reactors causes structural thermomechanical stresses and opposes the creation of natural convection essential for fulfilling safety as one of the GEN-IV roadmap objectives. Frignani et al. [78] improved the thermal-hydraulics of the ALFRED concept to provide an appropriate operational environment for research groups, and safety authorities in European Technology Demonstrator Reactor.

Schriener and El-Genk [79] looked at exploring the possibility of gas-lift pumping to increase the free flow of HLM for potential use in cooling advanced nuclear plants, as well as in other energy and industrial applications. A model based on the principles of thermal hydraulics was created to replicate the motion of liquid metals in the trial run. A two-part flow map was included in the model to compute the medium void fraction of the gas pumped in the riser parts of the trial circuit, which is efficient for improving the circulation of liquid metals. Additionally, [80] analysed the potential for implementing gas-lift pumping using the natural circulation loop to improve LBE natural circulation. The consequence of raising the riser height at the upper portion of the gas injection point on the LBE natural circulation was also studied and found to have improved the natural circulation.

Furthermore, the HORIZON2020 SESAME experimental campaign with major outputs of the ENEA’s NACIE-UP facility, in 2017 yielded post-processed data [81]. A standard thermal flow meter was used for mass flow rate measurement. Additionally, [82] investigated transient heat transfer thermo-fluid dynamic transients at the NACIE-UP site. As a result, a substantial amount of data was developed to aid the qualification of STH, CFD and coupled codes in lead-based fast reactors.

More so, investigations into heat transfer of a grid-spaced fuel assembly using liquid metal were done on the newly built ICE test portion at the CIRCE pool facility. The study also offers experimental data to enable the certification of codes for European fast reactor development [83]. It is reported that massive pool reactors have been used to research thermal stratification which is important for the design of HLM nuclear reactors, particularly in terms of safety.

At SCK-CEN, the E-SCAPE facility core simulator and LBE coolant give designers experimental input on forced and natural convection flow patterns [17]. The System and CFD approaches adopted in E-SCAPE and MYRRHA flow patterns demonstrated accurate performance.

### 4.3 System thermal hydraulics

A study on New Generation codes for ground-breaking projects has been conducted. The first was the creation of a set of codes of varying degrees of complexity that may be used to solve NPP safety duties using fast reactors constructed in Russia as part of the Federal Target Program for new-generation nuclear power technology [84]. The coding system enables a consistent multi-physics and multiscale examination of operational and emergency regimes to be carried out. Ahn et al. [85] report on the FONESYS code to support code users in the nuclear industry. As a result, they established a forum and network for programmers as users. However, meshless CFD algorithms based on Lagrangians have recently been created and are being employed in nuclear thermal hydraulics and safety applications [86]. Turbulent and multi-phase flows and heat transfer models developed based on the conservation equation are now included in the SOPHIA code.

THINS project was established to solve certain cross-cutting thermal-hydraulic challenges of several advanced nuclear technologies identified in Europe [25]. All test facilities participating in the project, except the ESCAPE facility, provided relevant test data necessary for advanced phenomena after four years. The extensive test database built for code qualification led to the creation of new correlations or models. Preliminary model assessments on the facilities were conducted using RELAP5 STH and the ANSYS CFX CFD code [18]. The pre-test study on the ESCAPE facility demonstrates the capability of reproducing the MYRRHA phenomena under forced and natural convection. However, a shift observed in ESCAPE under temporary conditions from forced to natural convection is different from that of MYRRHA.

Tarantino et al. [5] present their findings from the SESAME project, which aims to improve liquid metal coolant thermal hydraulics investigations in experiments as well as simulations. The aim of taking into account the system scale is to test and enhance STH models and codes as well as improve developmental approaches and validate multi-scale techniques. New reference data is being developed, both experimentally and numerically, to support not just the system scale and improve STH models and algorithms, but also to create and evaluate multi-level design methodologies. Analytical and empirical correlations of turbulent heat transport under various regimes of forced, mixed, as well as natural convection, were investigated and proven to solve thermal-hydraulic challenges.

Worthy of note is the upgrade of RELAP5/Mod3.3 code for lead-based coolants, which aids the ENEA in the LEADER project [87]. This upgrade allows for the assessment of the heat transmission of the CIRCE-HERO system. Several simulations were explored including loss of flow transients. The possibility of establishing natural circulation capable of secure removal of decay heat was evaluated in the steam generator of HERO. Similarly, [88] coupled RELAP5/Mod3.3 and FLUENT codes in the analysis of CIRCE-HERO STH. However, [89] use a coupled approach of FLUENT CFD code and RELAP5 STH code developed to demonstrate HX-HERO working conditions at the CIRCE facility at the University of Pisa. The numerical results produced a reliable temperature forecast by precisely predicting the temperature trend qualitatively and quantitatively. On the other breadth, [90] evaluated the CIRCE-HERO fundamental test facility's total LOF benchmark as part of a SESAME project. The numerical exercise revealed that the codes were capable of reproducing the key events encountered throughout the experiment.

Furthermore, an assessment was conducted on the NACIE loop by ENEA to aid the design and testing of the RELAP5/Mod3.3 STH code's new coupling method with the Fluent CFD model [91]. The coupling tool is used to model trials that mimic natural and isothermal gas-lift circulation phenomena. To overcome the shortcomings of separate use of STH and CFD approaches, their combination can help improve prediction capacity for each stand-alone application. Given that, [92] used the HYDRA-IBRAE/LM STH program to model lead/LBE coolant flow and performed validation of results. The experimental lead coolant study results from the Russian project PRORYV have been necessary for the assessment of STH codes. Code validation was then performed in a BREST-OD-300 design of a steam generator.

Forgione et al. [93] used STH codes to execute blind simulations of the NACIE-UP assessment activities. The results of experimental preliminary tests were used to adjust the computer models for system heat losses and better gas circulation. Also, [65] employed STH codes to conduct benchmark simulations of the HLM facility after tests to complete the system code creation, improvement, and validation. Four STH codes were revised to determine the potential numerical code to support the forecasting and reproduction of operational and inadvertent transients.

Van Tichelen et al. [94] conducted a thorough thermal-hydraulic analysis of occurrences in MYRRHA's engineering and safety assessments. They discovered that experimental programs support the validation of numerical tools and models in the well-established STH sector as well as in CFD. Therefore, more research and development are needed for design and licensing, including expert teams, the world over, as well as experimental facilities. Again, [19] noted that CFD simulations of a European-scaled pool facility can support the design as well as licensing of MYRRHA. Consequently, the CFD simulation results show the importance of CFD programs simulating the heat transfer of pool nuclear plants.

Additionally, [95] performed a validation of CFD assessment using data from ESCAPE pool experiments from the MYRTE project. NRG used the STAR-CCM + CFD code to create a model regarding the flow and transfer of heat in the ESCAPE pool under forced convection situations. The temperature distributions and heat loss of the pool were in good agreement with the measurements; however, the drop in pressure from the intake to the outlet was 20% higher than expected. This demonstrates the need for CFD software to accurately simulate pool reactors' thermal hydraulics and the importance of correcting geometrical errors in CFD models to accurately simulate pressure losses. Future modelling of flow and heat transmission in ESCAPE for natural convection and transient scenarios must be able to accurately simulate the pressure losses.

Zou et al. [96] performed a 3D thermal-hydraulic multi-scale coupling assessment of the top plenum in a pool-shaped LBE fast reactor employing the ATHLET-OpenFoam code. The ATHLET code reveals regions where stratification occurred. This suggests that the inlet's outer side vortex flow is changing at the outer area of the top plenum during the Unprotected-LOF.

Papukchiev and Buchholz [97] carried out validation of ANSYS CFX with conjugate heat transfer for flows involving gas as well as liquid metals. The TALL-3D experimental measurements of excellent quality and that of L-STAR support the acquisition of the numerical results. More so, in transient CFD simulations, [98] investigated the requirement for conjugate heat transfer modelling. The CFD simulations revealed that considering simply the fluid domain is insufficient for predicting the thermal-hydraulic transient dynamics correctly. Therefore, future simulations may take into account the thermal inertia and heat conduction of these components.

Testing the flow and heat transfer of a natural circulation loop with LBE coolant moving through a circular tube has been reported [99]. Theoretical research was done on the effects of roughness on HLM heat transmission, and preliminary findings were validated using Natural Circulation Capability Loop experimental data. The results demonstrate the important role of pipe roughness in the heat-transfer trials. Thus, more research into the flow characteristics of HLM is required to ascertain the effect of pipe roughness. More so, [23] carried out an experiment as well as a CFD benchmark of HLM natural circulation using a complete test loop to support SMRs. Using well-defined benchmark data, validation of the MARS-LBE STH code was performed. Based on experimental results in HELIOS, an empirical relation was created to forecast the mass flow rate for a non-isothermal, adiabatic state. Furthermore, experimental research is done on the heat transmission properties of an LBE in a vertical circular test section subjected to rolling and static motions [100]. To examine the heat transmission properties of LBE, a correlation is established under rolling states. The findings demonstrate that the rolling motion’s extra forces result in a periodic shift in the forced circulation's flow rate.

Instability in natural convection was studied and experimental data was produced to evaluate thermal hydraulics and CFD algorithms in standalone and coupled systems in an open and blind benchmark of TALL-3D [101]. As pre-test analysis is critical for selecting the experimental settings that will yield the most meaningful experimental data for code validation and benchmarking, [26] performed a pre-test study at the TALL-3D facility to identify natural circulation instabilities. The two main factors affecting the reliability of instability results are (i) secondary side modelling with assumptions on the efficiency of the heat exchanger and (ii) RELAP5’s axial heat conduction deficiency, and significant low flow rates.

In a pre-test study of unintentional transients for classification of the ALFRED SGBT mock-up, [102] explored the new idea of thermo-fluid behaviour and supplied some experimental data for the verification of STH code. Using 1-D modelling and 3-D CFD simulation, a transient examination of single-phase coupling was performed for the natural circulation loop system [103]. The transient research is crucial because it allows the development of heat exchangers that use buoyancy forces to perform heat transfer jobs. The study was to close this gap by employing a 1-D mathematical model to analyse the dynamic properties of the system. To fill this gap, detailed parametric model was examined with liquid sodium coolant.

Various reactors under construction including small, medium and large complex reactors use LBE coolant [79,104,105]. Nuclear reactors with improved natural circulation cooling can operate more quietly and safely. The simulation findings show that the LFR's compact modular natural circulation offers several major advantages, a good development option due to its reduced geometric design and excellent inherent performance. To analyse the complex thermal-hydraulic processes that take place in a typical pool-type reactor while also accelerating computation performance, more work will entail connecting system code and CFD code. Several validations of multi-scale approaches were performed at the TALL-3D facility in Sweden, employing coupled simulations and subsequent comparisons to experimental data from E-SCAPE [[106], [107], [108]]. The transient coupled simulation results for transition flow between forced and natural convection phenomenon is heavily influenced by flow mixing and stratification occurrences. Comparing data from 1D STH simulations with information from the natural circulation reveals that natural convection is influenced by complicated flow and temperature distributions and limits forecasting using 1D STH algorithms.

CFD makes it possible to analyse Generation IV nuclear reactors with high-quality 3D thermal hydraulics. However, to use the code in the decision-making process, it must be shown to be appropriate for the intended application. In a TALL-3D thermal hydraulic experiment using a liquid metal coolant, [68] validated the Star-CCM+ code. As sensitivity analysis is significant in the identification of the impact of important unknown input parameters on simulation outputs, solution verification is carried out during code validation to reduce numerical uncertainty. It was demonstrated that Star-CCM+ can predict well the effects of thermal loads in geometries. Again, [109] reviewed CFD and coupled system codes such as RELAP-3D that include 3D modules with limited capacity for 3D simulation. The review discussed the way validation and verification work for coupled codes as determined by the exchange of information between CFD and system codes.

Innovative Generation IV nuclear reactors require the use of the Best Estimate Plus Uncertainty (BEPU) technique in the advancement of knowledge and accurate assessment of safety margins for novel occurrences. RAVEN code was used to verify a method for uncertainty quantification (UQ) [110]. The efficacy of the embedded UQ method in a coupled calculation was examined using RELAP5-3D/RAVEN codes. The BEPU approach was demonstrated and deployed in a sophisticated LFR system that mimics the basic system.

Experiments that are required to gather data for any form of validation or benchmarking should have the same resolution as CFD as highlighted in standards and quality assurance for numerical simulations [42]. Therefore, the experiments should progress to achieve a more comprehensive level of detail, accuracy, and quantity of data as CFD resolution improves, in terms of space and time, as well as capturing an increasing number of physical events. Field values, in particular, are becoming increasingly important, and are difficult to obtain for liquid metal flows, coupled with determining highly resolved local flow variables with complete instrumentation. Relatively simple studies using liquid metals can be challenging since the true domain for exact placements of sensors is difficult to verify or the knowledge regarding the velocity field is not well understood.

Extensive experiments sometimes contain instrumentation that is too haphazardly distributed. As a result, benchmark campaigns for these experiments, such as plena, and sump cooling, coupled with containment flows, as well as those for small liquid metal flows, are once a while included in experiment evaluation workshops. Therefore, experimental data, particularly data from liquid metal flows, must be validated. For every new combination of the concerned physical configuration, as well as for every new prototypic flow event, detailed and highly instrumented tests are required coupled with the need for improved CFD user education, to validate relevant simplified physical problems relative to the numerical counterpart.

## 5 Key findings, achievements, and future directions in HLM-cooled reactors

### 5.1 Key review findings

This review document demonstrates the several research initiatives that have supported LFR design and development. Table 5 shows the key findings of this review work categorized under core, pool, and system thermal hydraulics and grouped under observation, conclusions, and recommendations.

**Table 5:** Key findings on core, pool and STH research activities.

Core Thermal Hydraulics	Pool Thermal Hydraulics	System Thermal Hydraulics
1) Observations: - CFD subchannel thermal hydraulics analyses tools and turbulence models are being developed for CFD modelling and simulation methodologies. - Heat transfer performance of wire-wrap and grid-spaced pin/bundles has been studied. - Simulation on vibration, deformation, blocked and unblocked fuel pin/bundle/assembly heat transfer analyses to be undertaken and validated. - Experiments reveal fluctuation regions exist behind the flow blockage zone.	- Heat exchanger experiments during the transition from forced convection to natural circulations revealed thermal stratification. - Gas-lift/injection in the riser was found to improve natural circulation. - Thermal stratification was seen in the top plenum of the pool-like design under various flow regimes. - Experimental data and correlations required for natural and transient circulation were performed on LOF accident.	- New correlations and models on heat transfer properties and turbulent non-dimensional parameters and algebraic heat flux models were produced based on the test database for model qualification. - Consideration of system scale required to test and enhance STH models and codes. - 3D thermal fluid-dynamic events have been seen and may affect how accidental transients like the LOF accident develop. - The significance of coupled simulation algorithms to model protected flow loss in internal and external loops was made clear. - Instability issues resulting from natural circulation experiments observed.
2) Conclusions: - Accurate prediction of coolant and cladding temperature is required. - Further study is needed on pressure-drop and turbulent models as well as heat transport correlations. - Detailed thermal hydraulic assessment is required for a wire-spaced bundle with variable flow rates and heating powers. - Transient thermal-hydraulic and evaluation LETHAC code developed and validated for lead-based fast reactors.	- Few studies carried out on pool-type reactor and steam generator tube rupture. - For safety, pool thermal stratification is important and analysis was performed and compared with experimental results. - Steam generator, protected loss of flow accident simulation analyses performed. - Experimental data is significant for CFD and system thermal-hydraulic code validation.	- Code coupling simulation improves safety analyses of pool-type reactors and needs to be studied further. - Conjugate heat transfer modelling is performed since CFD simulation in the fluid domain is insufficient for predicting thermal hydraulic transient correctly. - Meshless CFD codes based on Lagrangians using a smooth particle hydrodynamics approach developed. - Benchmarking using code coupling was performed that reproduces key events encountered in the experiment.
3) Recommendations: - Development of advanced turbulence models and numerical methodologies for evaluating differential pressure and heat characterisation in fuel bundles are needed. - Further studies on heat transfer and pressure loss to support small blockages and the effect of IWF between surrounding fuel assembly is needed. - Installation of thermocouples and heavy instrumentation of test section for analysis in different flow regimes to be carried out. - Performance of benchmark activities for code validation should be encouraged - Model experiments should be to comprehend physics, validate experimental instruments and qualify the design for licensing.	- CFD models for tracking steam bubbles in the Eulerian flow field to model bubble motion in the core to be developed. - Further study is required on the transmission of pressure waves and sloshing as well as the mechanical influence on structures in heat exchanger rupture accidents, which were modelled. - Future studies to include mixed convection and expansion of validity area for HLMs.	- There is the need to further develop and validate multi-scale techniques. - Utilization of CFD in circumstances containing 3D settings, such as a pool or bundles of wire-wrapped rods is required, where a lumped parameter approach to a system's thermal-hydraulic behaviour was inefficient for the most precise prediction realization. **-** Likelihood of employment of empirical formula in future studies in flow transients caused by changes in power level. - Use of BEPU code development for multipurpose and probabilistic and UQ required. - Performance of verification, validation and UQ and sensitivity analysis of unknown input parameters.

### 5.2 Achievements in LFR technology development

The main areas of advancement in the thermal-hydraulic exercise covered in this review paper are turbulent heat transport in the core, pool, and system of HLM nuclear reactors. Specifically, a few research achievements are in the following areas:

• Development and evaluation of heat flow models, algebraic heat flux models, as well as correlation;
• Development of modern experimental and efficient numerical data to validate the RANS model of HLM flow in wire-wrapped in addition to grid spacers fuel assemblies;
• Experimental and high-quality numerical reference values with an emphasis on jet, separation and rod bundle flow;
• The IWF validation using data from the experimental and numerical investigation;
• Creation of relevant quality experimental data through multi-scale tests to real reactor scale to validate HLM STH;
• Use of modern experimental data coupled with efficient numerical models to validate liquid metal coolant flow-induced vibrations in fuel assemblies;
• Expansion and creation of new experimental data for validation from e.g., TALL-3D, CIRCE and NACIE-UP facilities among others for HLM thermal hydraulics;
• Simulation of STH codes with coupled multi-scale tools for comparison with experimental outcomes to enhance the validation of numerical tools; and determination of modelling and measurement issues;
• Development of core, pool and system model validation using CFD tools.

The LFR technology development has seen significant progress in recent years. Several achievements have been made, including the development of advanced fuel assembly design, the demonstration of passive safety systems, the operation of an LBE-cooled reactor in normal and accident scenarios, and the completion of several experimental programs. Additionally, the development of innovative fast reactor cores and heat exchangers has been a major focus of research and development. This technology has seen great advances and is poised to make a major impact in the nuclear energy sector.

### 5.3 Key future research directions in LFR development

Although numerous OECD countries have done research in the vital areas of materials/corrosion and thermal-hydraulics of lead and LBE, there are still a few thermal hydraulic issues with liquid metal-cooled reactors [9]. For the readiness of LFR technology, the following future thermal hydraulic issues have been identified and are presented in this review paper:

• Upgrade of instrumentation and measurement techniques for accurate data acquisition;
• Refinement of fuel pin and assembly simulation codes for normal and off-normal scenarios;
• Development of heat transfer technology in terms of methods, models and codes for bubbly two-phase flow simulations;
• Encourage benchmarking for qualifying numerical codes for new single and integral effects experiments on relevant geometries and basic flow behaviours;
• Improvement of laminar or turbulent heat transmission correlations along thermally loaded surfaces;
• Enhancement of modelling and simulation technology for flow mixing in forced, transitional, and buoyant flow;
• Development of a DHR testing facility to enhance flow and heat transfer technology in the heat exchanger;
• Enhancement of gas-lift modelling and simulation;
• Development of sloshing and Seismic testing facility and Fluid-Structure Interactions (FSI) modelling techniques;
• Provide facility for analysing serious accidents and studying the causes of core damage events and support validation through experimental data;
• Development of new heat transfer correlations;
• Refine coupling codes for multi-scale and multi-physics thermal hydraulic analysis;
• Enhance the training of users on the development of modelling and simulation techniques;
• Providing appropriate guidelines to follow in model verification, validation and UQ.

Thermal hydraulics research of fast reactors employing HLM coolants involves investigating the flow characteristics, flow stability, turbulence, heat transfer and FSI of the coolant. It also involves assessing the performance of the fuel pin bundle and core, safety analysis and emergency core cooling, heat exchangers and pumps, flow-induced vibrations, mixing and stratification, corrosion and material compatibility, and containment confinement structures. These research areas are key to ensuring the safe and efficient operation of fast reactors.

## 6 Conclusion

This review paper discusses thermal-hydraulic operations that are supported by experimental facilities to improve the design and development of novel LFRs. A short description of some experimental facilities cooled by Lead/LBE that assist thermal hydraulics operations has been provided, starting with design concerns and goals. The experimental and numerical design and advancements in fast reactors have been published in the areas of the core, pool, and system thermal hydraulics as well as heat transfer modelling. The last 20 years' worth of LFRs' global accomplishments and upcoming difficulties have been compiled.

To validate the system, subchannel, and CFD codes for HLM systems, experimental data from a facility is required. Very little is known about the 3D simulation of the thermal-hydraulic behaviour of the LBE coolant in the transition flow regime. However, few studies were carried out in the areas of a gas lift, BFPS, forced and mixed convection, fuel pin bundle, and passive decay heat removal in natural circulation. Further research is needed in the areas of modelling and simulation, correlations and methods, material testing, models, and codes to enhance LMFR technology. The objectives of the individual or team research efforts in benchmarking are realized through the interdependence of experiments and numerical simulations.

A sustainable, secure, clean, and safe energy future will be ensured by this evaluation, which will expand knowledge, experimental facilities, and advanced nuclear reactor technology.

## Funding

There have been no grants advancement from any commercial, public or non-profit organization in funding and support of this research work.

## Author contribution statement

All authors listed have significantly contributed to the development and the writing of this article.

## Data availability statement

No data was used for the research described in the article.

## Declaration of competing interest

The authors declare that they have no known competing financial interests or personal relationships that could have appeared to influence the work reported in this paper
